# Nursing Leadership in a Rapidly Aging Society: Implications of “The Future of Nursing” Report in Japan

**DOI:** 10.1155/2012/820345

**Published:** 2012-06-27

**Authors:** Harue Masaki, Hiroko Nagae, Megumi Teshima, Shigeko Izumi

**Affiliations:** ^1^Graduate School of Nursing, Chiba University, 1-8-1 Inohana Chuo-ku, Chiba 260-8672, Japan; ^2^College of Nursing, Washington State University, P.O. Box 1495, Spokane, WA 99210-1495, USA

## Abstract

The recent US Institute of Medicine (IOM) report about the future of nursing highlights the areas where nurses can serve, contribute, and move forward to improve health care in the United States. Japanese nursing scholars examined the IOM report for its implications in the Japanese context and explored the future of nursing in Japan. The purpose of this paper is to provide support for the premise that the report's recommendations could have implications for the future of nursing outside of the United States, especially in Japan. Particular areas and activities by nurses in Japan will be presented as examples of nurses taking leadership in designing care for the rapidly aging society of Japan.

## 1. Introduction

The landmark report “The Future of Nursing: Leading Change, Advancing Health” by the US Institute of Medicine (IOM; [1]) should be read with keen interest not only by nurses and related professionals in the US, but also by health care professionals around the world. Japan is no exception. Although the health care system in Japan differs from the US system, Japan faces similar health care challenges as the US, which are delineated in the report. Therefore, the recommendations in the report are considered to have significant implications for the future of Japanese nursing as well. The purpose of this article is to provide support for the premise that the report's recommendations could have implications for the future of nursing worldwide, especially in Japan. Particular areas and activities by nurses in Japan will be presented as examples of nurses taking, or needing to take, leadership in designing care for the rapidly aging society in Japan.

## 2. The Japanese Context for the Future of Nursing

 The most prominent issue in the Japanese context that needs to be considered when thinking about health care in Japan and the future of nursing is that Japan has the fastest growing aging population in the world with an increasing number of deaths due to aging. The population aged 65 years or older increased from 7.4 million (7.1% of the total population) in 1970 to 29.4 million (23.1% of the total population) in 2010 [2]. Furthermore, it is estimated that, by 2050, more than one-third of the Japanese population will be 65 years and older [2]. Because the prevalence of chronic illness increases with age, growth in the elderly population results in increased demand for health care services and increased health care costs in Japan. Although Japan has had a relatively cost-effective national health care plan, this drastic increase in the elderly population and their needs for health care service will crash the current health care system in the near future [3, 4]. Furthermore, elderly patients tend to stay in hospitals longer due to their complex conditions with multiple comorbidities and frailty. In order to compensate the hospital cost exceeding the diagnosis-based reimbursement, physicians are forced to order treatments or examinations which they can charge but may not be necessary for the patients. This type of manipulation of the health care reimbursement system does not only distort the health care cost but also skew quality of care the patients receive in hospitals. Unfortunately, most of nurses in Japan are not involved to the financial decision making in their hospitals and rarely intervene to manage the balance between cost and quality of care to the patients.

The increase in the number of older people also means an increase in the number of deaths in society. The number of deaths is estimated to increase 170% within the next 30 years in Japan. In 2009, 81.1% of deaths in Japan occurred in hospitals, and most older adults who die in hospitals are admitted from home when their conditions deteriorated and care at home by family members became difficult; they are not admitted to receive medical treatment [5]. Japanese hospitals are primarily designed to provide medical treatment to acutely ill patients, not to provide quality end-of-life care which requires sufficient time and care and less medical intervention. Hospitals, particularly small size community hospitals, in Japan include certain number of “long-term care beds” in their facilities to accommodate older adult patients who need care for prolonged deteriorating conditions and end-of-life. However, in hospital settings where acute care precedes to nonrevenue generating long-term care, staffing and services they provide to patients in the “long-term care beds” are inadequate. In addition, it will become impossible when the number of older patients who need that type of care increases as estimated. Furthermore, to contain ever-rising health care cost and to improve efficiency of health care delivery system, in 2005, the Ministry of Health, Labor, and Welfare in Japan proposed to reduce significant number of long-term care beds while increase the number of health care services to provide care in community [6]. Unfortunately, the planned transformation of health care delivery system is stagnant due to the lack of concrete strategies and effective incentives to expand services to care for older adults in their community.

These challenges ask for innovative reform of the health care system and a concomitant transformation of the nursing profession. Similar to the IOM report in the US, the primary concerns driving health care reform in Japan are quality, access, and value/cost of care. The fundamental principles identified in the IOM report to guide changes to meet the challenges are thought to be applicable to the Japanese context. The principles include more patient-centered, primary care and prevention than highly specialized medical treatment, more care delivered within community settings than hospitals, and coordinated, seamless care across health conditions, settings, and providers [1].

## 3. Exemplary Activities by Nurses to Promote Change in Health Care in Japan

 A group of nurses led by a Certified Gerontology Nurse Specialist (CNS; an advanced practice nurse similar to a Clinical Nurse Specialist in the US) in a hospital demonstrated their leadership in promoting change to improve end-of-life care for the growing number of older adult patients in their hospital and the community [7]. In their mid-size, acute care community hospital, it was common to provide all treatments the hospital is equipped to provide regardless of patient's age, general health status, and their wishes. Because it is difficult to determine the prognosis of older adult patients with multiple chronic illnesses, the nurses observed that many older adult patients spent their last days and weeks of life in the hospital receiving unnecessary or ineffective medical treatment, suffering from pain and lack of dignity. In order to provide adequate care for these older adult patients, the nurses in this hospital started in-hospital conferences to share information about patients' preferences about end-of-life care and discuss the quality of life of each patient. By involving nurses, physicians, social workers, nutritionists, and physical therapists in the hospital and care managers in the community in the discussions, the nurses encouraged reflection on their values and shifted the culture of the hospital from medical treatment-focused to patient-centered care. Furthermore, the nurses started community services facilitating discussion about and preparation for better end-of-life care among the elderly as part of a healthy aging class in their community.

 This series of activities led by the CNS was reported as part of a symposium at the annual meeting of Japan Academy of Gerontological Nursing [7]. Although no hard evidence to demonstrate improvements in quality, access, or cost was presented at the conference, the positive changes this CNS created were noticeable. Not only were each patient's wishes and preferences respected in the hospital, people in the community had opportunities to think about their end of life, were educated about options for end-of-life care, and were prepared to prevent unnecessary or unwanted burden and suffering. This group of nurses embodies all of the principles of patient-centered care, primary care, delivery of care in the community, and coordination of care across settings and providers. In addition, by including physicians and other health care team members in discussions and reflections, the nurses were successful to build a collaborative team acknowledging each discipline's values while focusing on patients, not their discipline.

 Another exemplary work was led by certified advanced practice nurses working in a hospital. Their work was noticed by one of the authors (H. Nagae) when she was involved in a project about promotion of home-visiting care. The rapid increase in the older adult population and the number of people who require health care results in growing needs for places where frail older adults with chronic health problems can live safely while receiving continuous care. A new long-term care insurance system for this population was in place in 2000 as a government policy. The long-term care health policy emphasizes the importance of home care rather than institutional care as a solution for reducing medical costs. Home care in Japan has developed over the past 40 years, and nurses have taken leadership roles in promoting home care [8]. However, despite the emphasis on the importance of home care, the number of home care services (called Visiting Nurse Stations) has not increased over the last ten years because of a shortage of home-visiting nurses. More than 87% of nurses in Japan work in hospital settings and less than 10% work in community, home-visiting, or long-term care facilities [9]. Most of nursing education is focusing acute care, and few courses teach skills and knowledge necessary to work in home-visiting settings [10]. While patients in home care settings are aging and have more serious conditions with complications, fewer immediate supports are available for home-visiting nurses to manage complex condition of a patient in home care [11]. Therefore, higher competency and ability to work independently are required for home-visiting nurses. Fifty-five percent (55%) of Visiting Nurse Stations are small in size (<5 employed nurses) [10], and the reimbursement is controlled tightly to minimal cost, and there is no financial incentives to improve quality of care or provide continuous education for nursing staffs [12]. A lack of supporting resources and perceived heavier responsibility make many nurses, particularly newly graduated nurses, reluctant to pursue careers in home-visiting nursing.

 In order to facilitate early discharge and a smooth transition from hospital to home care, a group of certified nurses (CNs) in a hospital started care meetings with nurses working in local Visiting Nurse Stations. In the meetings, information about patients who were to receive care from the visiting nurses at their homes after being discharged from the hospital was shared. However, it soon became apparent to the CNs that the home-visiting nurses needed to acquire new knowledge and skills about how to take care of the various medications and new medical equipment that many patients would need to use at home. Because many Visiting Nurse Stations are small, they often do not have resources to offer continuing education to their home-visiting nurses to update their knowledge and skills about new drugs and medical technologies. The CNs in the hospital now offer in-service education about drugs and new equipments and consultations to home-visiting nurses during their meetings.

 This is another example of nurses' innovative activities beyond the boundaries of the care setting to improve quality, access, and cost of health care in an aging society. Several studies have reported that the major determinant of the place of care or place of death for terminally ill patients is the availability of home-visiting care by nurses and physicians [13–15]. If there are enough home-visiting nurses who are knowledgeable about how to use various drugs and new medical technologies that the patients bring home, the number of patients who can go home with home-visiting care will increase. In addition, the number of re-hospitalizations due to mismanagement of the equipment or inadequate symptom control will decrease. Another factor that increases patients' chances of dying at home as they wish is early introduction and transfer from hospital to home care [16, 17]. By developing a regular collaborative relationship between hospital nurses and home-visiting nurses, it becomes possible to make an early discharge plan, coordinate smooth transition, and provide continuous care from the hospital to home. In addition, home-visiting nurses are often familiar with local general practitioners who are supportive and available for home end-of-life care [18]. They can provide critical and useful information to support patients having access to appropriate care while they are at home.

 This kind of collaborative work across settings and providers is critical to expanding the care options that patients can choose and to providing seamless care through their illness trajectory. The work of these nurses represents principles of patient-centered care, the linkage between specialty care in hospital and primary care in the community, and collaboration and coordination across settings and disciplines.

## 4. Discussion, Limitations, and Future Directions of Nursing Leadership in a Rapidly Aging Society

 Two exemplary works by Japanese nurses were presented to illustrate the implications of the IOM report “The Future of Nursing” in the Japanese context. As seen in these works, nursing has enormous potential to take a lead in the reconstruction of health care in the face of the challenges posed by the rapid aging of society in Japan. However, we also want to point out some strengths and weaknesses in these practices.

First, in order to make these nurses' activities socially visible and to convince society, the nurses' works need to be disseminated with evidence. The first example was presented at a symposium during a nursing conference. It is inspiring for nurses to see their colleagues' innovative activity and its success. However, their work needs to be disseminated to other health care disciplines, hospital administrators, and the public, including the general public, public health workers, and local policy makers. The impact of their activities on patients and their families, the hospital, and the local community needs to be evaluated and presented to the public as evidence of the need to improve care and promote health care modification. Second example of collaborative work by CNs and home-visiting nurses has not yet been reported anywhere. Collaborative meetings of hospital nurses and home-visiting nurses might start from the good will of the nurses involved. But in order to sustain the activity and promote more systematic health care change, their work needs to be reported and evaluated. In our understanding, there are many innovative practices by nurses that are unfortunately not well evaluated or well known. To make the nurses' work more visible and for nurses to take a leadership role in health care reform, more rigorous efforts to improve data collection, analysis, and dissemination of nurses' practices are necessary. The IOM report makes this point clear as their recommendation.

It is interesting to see that both examples were led by advanced practice nurses such as CNSs and CNs. Seeing nurses with advanced degrees or advanced practice certificates demonstrate such leadership to promote change is encouraging. We want to point out that CNs have a minimum of 6 months of education in their specialty area and 5 years of nursing experience in addition to their registered nurse license, while all CNSs have master's degrees in nursing. What we learn from the work by the CNs in this example is that the length of formal education or the advanced degree may not be what is necessary to building leadership skills. Leadership styles may vary among individual nurses and the cultures where the nurses practice. Identifying effective leadership styles and skills required by nurses to make changes in Japanese society as well as methods for teaching leadership are necessary next steps. In addition to the IOM report emphasizing the importance of progressing nursing degree from licensed practical nurse (LPN) to the associate's, bachelor's, and master's of science in nursing, and to the PhD and doctor of nursing practice, we are contemplating the possibility to acknowledge and reinforce the nurses' experiential learning in their practice outside of formal education programs. In two examples seen above, the nurses who initiated and lead the activities had an advance degree or a certificate in their specialty. However, in collaboration with these advance practice nurses, many other nurses who do not have advance degree also demonstrated their expertise in their practice settings and contributed to promote effective changes. Because the nurses without advance degree are still the majority of practicing nurses, we should think about utilizing their expertise and practical wisdoms to promote changes in wider range of settings and various levels of practice.

The IOM report “The Future of Nursing” highlights many areas where nursing can serve and take leadership to move forward in the context of the US health care system. Since Japan faces unprecedented challenges to provide quality care to an increasing number of elderly persons while controlling care costs, Japanese nurses can learn from the IOM report because both countries are facing somewhat similar challenges. Many countries are facing or soon will face similar challenges. As we learn from the IOM report and examine our nurses' practices to foresee the future direction of nursing in our country, nurses in other countries can also learn from each other. Although careful examination in the context of each country is necessary, the IOM report provides overall direction for the future of nursing in many other countries.
